# Acute Responses to Different Velocity Loss Thresholds during Squat Exercise with Blood-Flow Restriction in Strength-Trained Men

**DOI:** 10.3390/sports12060171

**Published:** 2024-06-20

**Authors:** Juan Sánchez-Valdepeñas, Pedro J. Cornejo-Daza, Luis Rodiles-Guerrero, Jose A. Páez-Maldonado, Miguel Sánchez-Moreno, Beatriz Bachero-Mena, Eduardo Saez de Villarreal, Fernando Pareja-Blanco

**Affiliations:** 1 Science Based Training Research Group, Department of Sports and Computer Sciences, Universidad Pablo de Olavide, 41013 Seville, Spain; pjcordaz@upo.es (P.J.C.-D.); lrodiles@us.es (L.R.-G.); joseapm@euosuna.org (J.A.P.-M.); msmoreno@us.es (M.S.-M.); bbachero@us.es (B.B.-M.); fparbla@upo.es (F.P.-B.); 2 Faculty of Sport Sciences, Department of Sports and Computer Sciences, Universidad Pablo de Olavide, 41013 Seville, Spain; esaesae@upo.es; 3 Department of Human Movement and Sport Performance, University of Seville, 41013 Seville, Spain; 4 Centre Attached to the University of Seville, University of Osuna, 41640 Osuna, Spain; 5 Department of Physical Education and Sports, University of Seville, 41013 Seville, Spain

**Keywords:** resistance training, fatigue, neuromuscular, tensiomyography, metabolic response

## Abstract

(1) Background: The aim of this paper is to analyze the acute effects of different velocity loss (VL) thresholds during a full squat (SQ) with blood-flow restriction (BFR) on strength performance, neuromuscular activity, metabolic response, and muscle contractile properties. (2) Methods: Twenty strength-trained men performed four protocols that differed in the VL achieved within the set (BFR0: 0% VL; BFR10: 10% VL; BFR20: 20% VL; and BFR40: 40% VL). The relative intensity (60% 1RM), recovery between sets (2 min), number of sets (3), and level of BFR (50% of arterial occlusion pressure) were matched between protocols. Tensiomyography (TMG), blood lactate, countermovement jump (CMJ), maximal voluntary isometric SQ contraction (MVIC), and performance with the absolute load required to achieve 1 m·s^−1^ at baseline measurements in SQ were assessed before and after the protocols. (3) Results: BFR40 resulted in higher EMG alterations during and after exercise than the other protocols (*p* < 0.05). BFR40 also induced greater impairments in TMG-derived variables and BFR10 decreased contraction time. Higher blood lactate concentrations were found as the VL within the set increased. BFR0 and BFR10 showed significantly increased median frequencies in post-exercise MVIC. (4) Conclusions: High VL thresholds (BFR40) accentuated metabolic and neuromuscular stress, and produced increased alterations in muscles’ mechanical properties. Low VL could potentiate post-exercise neuromuscular activity and muscle contractile properties.

## 1. Introduction

Resistance exercise with blood-flow restriction (BFR-RE) implementation consists of placing an inflated pneumatic cuff on the most proximal portion of the exercising limbs. This action leads to a reduction in arterial blood inflow and total occlusion of venous return [1]. Consequently, this methodology triggers a localized hypoxia process within muscle tissue [2]. In response to this localized environment, BFR-RE induces greater adaptations in both strength and hypertrophy compared to low-load resistance training (RT) without flow restriction [3,4]. However, while BFR-RE yields muscle mass gains comparable to those produced by free-flow high-load RT, it seems to result in more modest improvements in muscle strength [5,6].

When implementing BFR-RE, consideration must be given to methodological aspects, including cuff width [7], the level of applied pressure [8], expressed as a percentage of the arterial occlusion pressure (AOP), and the duration of pressure application, whether it is continuous or intermittent throughout the training session [9]. Additionally, BFR-RE typically involves the use of light loads [20–30% of one-repetition maximum (1RM)], coupled with a high volume of repetitions per set conducted to failure [10] or close to it [11,12]. The latter characteristics make BFR-RE an effective method to promote muscle growth but it may not be as effective for developing maximal strength [5,6]. These are thought to be the main mechanisms that promote muscle development after BFR-RT: 1) increased metabolic stress [13], muscle fiber recruitment [3], cellular swelling [14], increased intramuscular signaling for protein synthesis [15], and myogenic stem cell proliferation [16]. It has also been suggested that BFR-induced muscle hypertrophy involves not only the proliferation of contractile proteins but also an increase in muscle glycogen stores [17] and increased capillary number [18].

When performing each repetition at maximal voluntary effort, the force applied and, hence, the lifting velocity gradually decrease due to fatigue development [19]. In this context, velocity loss (VL) within the set has emerged as an objective, practical, and non-invasive marker of fatigue during RT [20]. This statement is based on the close correlations (R^2^ = 0.92–0.97) observed between the achieved VL within the set and the percentage of completed repetitions relative to the maximum possible [21,22]. Specifically for the full-squat (SQ) exercise, when 50% of possible repetitions are completed, it has been suggested that VL is approximately 20%, while when repetitions are performed close to failure, VL is around 40–50% [20,21]. This fact is relevant for load control, as individuals may not perform the same number of repetitions at a certain %1RM, yet they would exert a comparable level of effort (i.e., proximity to failure) with a given VL. Acute investigations have demonstrated that RT under distinct VL thresholds induces differing levels of fatigue, leading to variations in post-exercise recovery of mechanical and neuromuscular parameters, such as countermovement jump (CMJ) height, force, velocity, power output, and muscle activity [23]. Regarding long-term adaptations, high VL thresholds (≥40%) maximize muscle hypertrophy [24,25,26]; however, these thresholds may also induce adverse neuromuscular adaptations [25]. Conversely, moderate VL thresholds (i.e., 10–20%) seem to maximize strength gains [24,25,26]. It has been demonstrated that greater VL thresholds do not yield further 1RM strength gains compared to lower thresholds and may even lead to diminished strength gains, particularly in high-velocity actions [24,25,26].

Research indicates that achieving high levels of fatigue during free-flow RE is suboptimal for maximizing strength gains [24,25,26]. However, it is noteworthy that BFR-RE is normally conducted to failure [10] or close to it [11,12]. This practice leads to exceptionally high levels of fatigue, potentially causing counterproductive effects on specific neuromuscular adaptations. Moreover, VL within the set has been suggested as an indicator of fatigue development during RE [20,21,22]. However, no previous study has explored the use of VL for quantifying fatigue in the context of BFR-RE. Therefore, the purpose of this study was to analyze the acute effects of different VL thresholds during SQ with BFR on strength performance, neuromuscular activity, metabolic response, and muscle contractile properties.

## 2. Materials and Methods

### 2.1. Study Design

**Figure 1:**
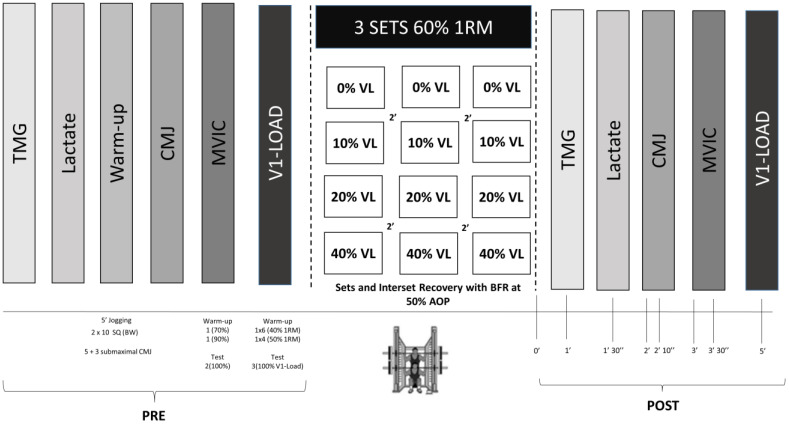
Schematic representation of the study design including the timeline of the battery of tests before and after the four performed protocols. TMG: tensiomyography; SQ: full squat; BW: body weight; CMJ: countermovement jump; MVIC: maximal voluntary isometric contraction; V1-load: load that elicited a 1 m·s^−1^ at Pre-test (~60% 1RM); 1RM: one-repetition maximum; VL: velocity loss magnitude scheduled within the set; BFR: blood-flow restriction; AOP: arterial occlusion pressure.

A randomized cross-over trial was conducted to analyze the acute effects of VL attained within the set in a BFR-RE context on the mechanical, metabolic, neuromuscular, and muscle contractile responses. Subjects performed four SQ protocols with BFR that differed in the VL attained within the set (BFR0: 0% VL, BFR10: 10% VL, BFR20: 20% VL, and BFR40: 40% VL). The relative intensity (60% 1RM), inter-set recovery (2 min), number of sets (3), and the BFR pressure (50% AOP) were matched between protocols. The protocols were conducted in a randomized order separated by at least 96 h. A battery of tests was performed before (Pre) and after (Post) each protocol, in this order: (a) tensiomyography (TMG), (b) blood lactate, (c) CMJ, (d) maximal voluntary isometric contraction (MVIC) in 90° SQ, and (e) performance with the load that elicited a ~1 m·s^−1^ velocity at baseline measurements (V1-load) in SQ (Figure 1). Moreover, to compare the performances obtained throughout the session, mean propulsive values of force (MPF), velocity (MPV), and power (MPP) along with electromyography (EMG) values were recorded for each repetition. Subjects were requested to refrain from engaging in any other strenuous physical activities for 72 h preceding each protocol. A week before starting this study, the AOP was determined and a progressive loading SQ test was conducted to calculate the individual load–velocity relationship in the SQ exercise. Testing procedures were performed in a research laboratory under the direct supervision of the researchers, at consistent times of day for each subject (±1 h) and in similar environmental conditions (20 °C and 60% humidity).

### 2.2. Subjects

Twenty moderately resistance-trained men (age: 24.5 ± 4.8 years; height: 1.79 ± 0.07 m; body mass: 76.3 ± 8.6 kg) participated in this study (tiers 1 and 2, as per the framework outlined by [27]). All subjects possessed at least one year of SQ training background (1RM relative to body mass: 1.49 ± 0.26) and two months of BFR-RT experience. Before starting the intervention, subjects signed a written informed consent after being informed about the risks of the protocols. No physical limitations, health concerns, or musculoskeletal injuries that could impact the testing were reported among the subjects. Subjects were discouraged from taking any supplement or drug that could disturb the results and to vary their daily diet during the intervention. The present study was approved by the Research Ethics Committee of “Hospitales Universitarios Virgen Macarena-Virgen del Rocío” (Reference: 1547-N-19), in accordance with the Declaration of Helsinki.

### 2.3. Testing Procedures

#### 2.3.1. Arterial Occlusion Pressure

A cuff (12 cm wide × 86 cm length; VBM20-54-528, Medizintechnik, GmbH, Sulz aN, Germany) was placed on each thigh as proximal as possible to the groin. Subjects remained seated for 10 min and with their knees flexed at 90°. The pulse was detected using a hand-held Doppler probe (Dopplex D900, Huntleigh Healthcare Ltd., Cardiff, UK) set on the posterior tibialis artery. The cuff was manually inflated using a hand-operated inflator (VBM20-18-601-VBM20-18-602, Medizintechnik, GmbH, Sulz aN, Germany) until the pulse was no longer present [28]. The lowest cuff pressure at which the pulse was no longer detectable was determined as the AOP. This process was repeated twice for both legs, with a 5 min rest interval between attempts. If the difference between each attempt was higher than 20 mmHg, a third attempt was performed. The mean AOP of the 2 attempts with each leg was recorded for further analysis. For the right and left legs, the AOP was 242.6 ± 21.1 and 242.0 ± 19.7 mmHg, respectively.

#### 2.3.2. Progressive Loading Test

The individual load–velocity relationship and 1RM load in SQ were determined on a Smith machine (Multipower Fitness Line, Peroga, Murcia, Spain) with no counterweight mechanism. Subjects began from an upright position with fully extended knees and hips, adopting a stance approximately shoulder-width apart, and positioning the bar across the back at the level of the acromion. While the eccentric phase was performed in a controlled manner, participants were instructed to execute the concentric phase at maximal velocity for each repetition. All repetitions were recorded with a linear velocity transducer (T-Force System Ergotech, Murcia, Spain), which is highly reliable [20]. The warm-up comprised 5 min of jogging at a self-selected easy pace, succeeded by 2 sets of 10 squats performed without external load, followed by 6 repetitions using a 20 kg load on the Smith machine. The initial testing load was 20 kg and was progressively increased by 10 kg increments (reduced to 5 kg, then 2.5 kg when the MPV dropped below 0.60 m·s^−1^) until the MPV reached ≤0.5 m·s^−1^. Three repetitions were performed with light (≥1.00 m·s^−1^), two with medium (1.00–0.80 m·s^−1^), and only one with the heaviest loads (≤0.80 m·s^−1^). Inter-set recoveries lasted 3 min, and only the repetition with the highest MPV for each load was recorded for further analysis.

#### 2.3.3. Resistance Exercise Protocol

Figure 1 depicts a timeline of the protocols. Before starting the test, subjects remained supine for 10 min to mitigate the potential effects of any preceding activity. While subjects were resting, TMG and EMG electrode marks were set, and resting blood lactate concentration was obtained. After TMG measurements were carried out, CMJ and MVIC tests were conducted. Then, a standardized warm-up was performed, consisting of 6-6-4 repetitions with 20 kg, 40%, and 50% 1RM, respectively, with a 3 min rest interval between sets. The relative loads were determined based on the MPV achieved for each %1RM, derived from the individual second-order load–velocity relationship (R^2^ = 0.995 ± 0.002) established during the progressive loading test. After the warm-up, 3 repetitions with the V1-load were performed (~60% 1RM), serving as the baseline dynamic strength measure. Afterward, the corresponding protocol was performed. All protocols were matched in intensity (60% 1RM), inter-set recovery (2 min), number of sets (3), and BFR pressure (50% AOP). The difference between protocols was the VL attained within the set (i.e., 0%, 10%, 20%, and 40%). Therefore, subjects performed repetitions until they attained the scheduled VL threshold in each set. BFR0 only performed one repetition per set to minimize the induction of fatigue. Throughout all protocols, strong verbal encouragement was consistently provided to motivate subjects to exert maximal effort. The BFR pressure (50% AOP) was established 30 s before starting the first set while the subject was seated in a chair and was maintained during the rest intervals to ensure a continuous restriction [10] within normal restriction times in BFR studies (5–10 min) [9]. Subjects had to be seated between each set so that the established pressure was maintained [28]. The SQ execution technique remained consistent with the description provided in the “progressive loading test” section. A force platform (FP-500, Ergotech) synchronized with a linear velocity transducer (T-Force System, Ergotech) was installed on the Smith machine to record MPF, MPP, and MPV for each repetition. Additionally, EMG data (i.e., RMS: root mean square; MDF: median frequency) were continuously recorded throughout the protocol. After the last repetition of the third set, the battery of tests was repeated as follows (Post): (1) TMG (at 1 min); (2) blood sample (at 1 min and 30 s); (3) CMJ (at 2 min); (4) MVIC (at 3 min); (5) V1-load (at 5 min).

#### 2.3.4. Tensiomyography

Contractile properties of the vastus lateralis muscle were measured with a TMG device (TMG-100 System electroestimulator, TMG-BMC, Ljubljana, Slovenia). Measurements were conducted with the subjects in the supine position, with the knee joint fixed at an angle of ~140° using a wedge cushion. Electric stimulation was administered through 2 self-adhesive electrodes (5 × 5 cm, Dura-Stick premium; Cefar-Compex, Hanover, Germany) positioned at a 5 cm interelectrode distance on the vastus lateralis muscle of the left leg, following SENIAM guidelines [29]. The muscle’s mechanical response was evaluated using a digital Dc-Dc transducer TransTekR (GK 40, Ljubliana, Slovenia) located equidistantly from the electrodes (25 mm) and perpendicular to the muscle belly. The measurements started with an initial current amplitude of 40 mA and a pulse duration of 1 ms, increasing 10 by 10 mA every 10 s until reaching the stimulator’s maximal output (100 mA) [30]. The variables analyzed were as follows: (a) the maximum radial displacement of the muscle belly (Dm), contraction time (Tc), delay time (Td), and velocity of deformation (Vd). Dm was defined as the peak amplitude in the displacement–time curve of the twitch response; Tc was obtained by determining the time interval from 10 to 90% of Dm; Td was defined as the time between the electrical stimulus and 10% of Dm [31]; and Vd was calculated as follows: Dm (Tc + Td)^−1^ [32]. All measurements were conducted by the same evaluator, and only the curve with the highest Dm value was selected for further analysis.

#### 2.3.5. Blood Lactate

After cleaning the skin, a blood capillary sample was collected using fingertip punctures on the middle finger. Lactate was measured using a portable lactate analyzer (Lactate Pro 2, Arkray, Kyoto, Japan). The reproducibility of this analyzer has been previously established within a physiological range from 1.0 to 1.8 mmol·L^−1^ [33].

#### 2.3.6. Countermovement Jump

Jump height was estimated from flight time using an infrared timing system (OptojumpNext, Microgate, Bolzano, Italy). Two attempts separated by a 10 s rest were completed, and the average height was calculated for further analyses. Subjects were instructed to perform the CMJ by resting both hands on their waist, executing a downward movement to about 90° of knee flexion, and trying to attain their maximal jump height. All subjects received instructions to land in an upright position and to flex their knees upon landing. A standardized warm-up was carried out, comprising 5 min of jogging, followed by 2 sets of 10 SQ without external load, and, subsequently, 2 sets of 5 and 3 submaximal CMJs, respectively.

#### 2.3.7. Maximal Voluntary Isometric Contraction

This test consisted of performing two attempts of 5 s maximal isometric SQ at 90° of knee flexion, measured using a handheld goniometer. The attempts were separated by a 30 s rest period. The test was conducted on the same Smith machine used in previous measures with height-adjustable movable supports. The warm-up protocol comprised 2 attempts at 70 and 90% of the maximal perceived effort, with a 30 s rest interval between them. Subjects were asked to push as hard and as fast as possible against the bar for 5 s upon the cue “Ready, set, go!” External forces were recorded at a sampling rate of 1000 Hz using an 80 × 80 cm dynamometric platform (FP-500, Ergotech, Murcia, Spain) and analyzed with specific software (3.65.1, T-Force System, Ergotech, Murcia, Spain). Maximal isometric force (MIF) and maximal rate of force development (RFDmax), calculated as the maximum slope in the force–time curve in 20 ms time intervals, were determined. EMG data were recorded as described below. The average value of the two attempts for each variable was calculated for subsequent analysis.

#### 2.3.8. V1-Load Test

Subjects performed 3 repetitions with the V1-load, which was determined as the load eliciting a 1 m·s^−1^ at the Pre-test (~60% 1RM) [20]. The SQ execution technique remained consistent with the description provided in the “progressive loading test” section. EMG data (i.e., RMS and MDF) were measured as described below. MPF, MPP, and MPV values were recorded with a linear velocity transducer synchronized with a dynamometric platform (3.65.1, T-Force System, Ergotech, Murcia, Spain). The highest value of each variable was saved for subsequent analysis.

#### 2.3.9. Electromyography Signal Acquisition

Following SENIAM recommendations [29] and after skin preparation, surface EMG electrodes were located in the vastus lateralis and vastus medialis muscles. EMG signals were recorded utilizing a parallel-bar, bipolar surface electromyography sensor Trigno wireless EMG system, featuring an inter-electrode distance of 10 mm, a common mode rejection ratio exceeding 80 dB, and a bandwidth filter ranging between 20 and 450 Hz ± 10% (Legacy, Delsys Inc., Natick, MA, USA). The baseline noise was maintained below 5 μV peak to peak, and the sampling rate was 2000 Hz. The raw EMG data were stored digitally using EMG Works Acquisition software (4.2, Delsys Inc., Natrick, MA, USA). During each contraction, the highest RMS and MDF values for each muscle were recorded over sliding windows of 500 ms with an overlap of 499 ms. The signal obtained from MVIC at the Pre-test of each protocol was employed to normalize the EMG parameters. The RMS and MDF values from both muscles were averaged for subsequent analysis.

### 2.4. Statistical Analyses

Data are reported as mean ± standard deviation (SD). Test–retest reliability was calculated by the coefficient of variation (CV) and the intraclass correlation coefficient with a 95% confidence interval [ICC (95% CI)], using the one-way random effects model. The Shapiro–Wilk test of normality was conducted to ensure normal data distribution at Pre. A repeated measures ANOVA was performed to compare differences between protocols within the training session. A 4 (protocol) × 2 (Pre vs. Post) repeated measures ANOVA was conducted to analyze the acute responses. Bonferroni’s post hoc adjustments were conducted when corresponding. Statistical significance was established at the *p* ≤ 0.05 level. Pre–Post effect size (ES) values were calculated using Hedge’s g on the pooled SD [34]. Statistical analyses were performed using SPSS software version 20.0 (SPSS, Inc., Chicago, IL, USA), and Microsoft Office Excel 2007 for calculating ES and CV. Figures were designed using GraphPad Prism version 8.0.0 (GraphPad Software, San Diego, CA, USA).

## 3. Results

The reliability values of the different tests conducted were as follows: CV values [Dm: 4.6%, Tc: 3.8%, Td: 3.4%, and Vd: 5.1%; CMJ: 1.7%; MIF: 6.9% and RFDmax: 16.2%; MPF: 3.6%, MPV: 5.3%, and MPP: 7.5%; RMS: 10.5% and MDF: 6.2%]; and ICC (95% CI) values [Dm: 0.99 (0.99–0.99), Tc: 0.97 (0.96–0.98), Td: 0.95 (0.92–0.97), and Vd: 0.98 (0.98–0.99); CMJ: 1.00 (0.99–1.00); MIF: 0.90 (0.85–0.94) and RFDmax: 0.85 (0.75–0.91); MPF: 0.98 (0.97–0.99), MPV: 0.88 (0.82–0.92), and MPP: 0.89 (0.84–0.93); RMS: 0.97 (0.96–0.98) and MDF: 0.95 (0.92–0.97)].

### 3.1. Descriptive Characteristics of the Resistance Exercise Protocols

Table 1 shows the main mechanical and neuromuscular characteristics of each resistance training protocol. As VL increased, the total repetitions and the average repetitions per set significantly increased but MPF, MPP, and MPV decreased (*p* < 0.05). Regarding neuromuscular variables, BFR40 evoked higher RMS and lower MDF than the other protocols (*p* < 0.05).

### 3.2. Tensiomyography

TMG-derived variables are reported in Table 2. BFR40 induced higher Dm and Vd decrements than the other protocols (“protocol × time” interactions: *p* = 0.03 and 0.01, respectively). Moreover, Tc was significantly decreased only after BFR10 (“protocol × time” interaction: *p* = 0.02). No significant “protocol × time” interaction was noticed for Td (*p* = 0.08).

### 3.3. Blood Lactate

**Figure 2:**
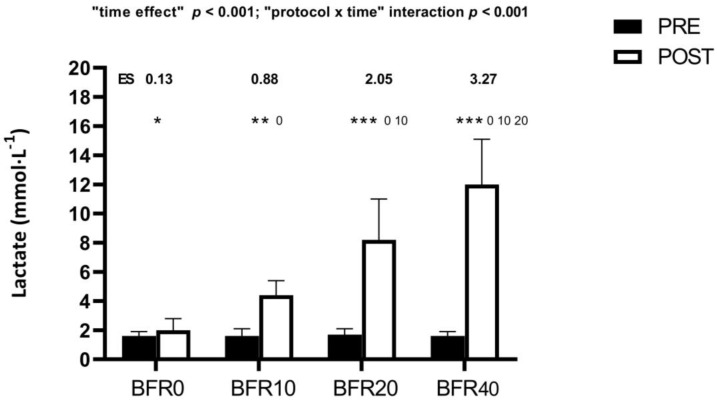
Blood lactate concentration following different resistance exercise protocols with distinct velocity loss thresholds during full-squat training with blood-flow restriction. BFR0: protocol with a velocity loss of 0% within the set; BFR10: protocol with a velocity loss of 10% in each set; BFR20: protocol with a velocity loss of 20% in each set; BFR40: protocol with a velocity loss of 40% in each set; ES: within-group effect size from Pre- to Post protocol; intragroup significant differences from Pre- to Post protocol: * *p* < 0.05, ** *p* < 0.01, *** *p* < 0.001. ^0^ Statistically significant differences with BFR0 protocol: *p* < 0.05. ^10^ Statistically significant differences with BFR10 protocol: *p* < 0.05. ^20^ Statistically significant differences with BFR20 protocol: *p* < 0.05.

A significant “protocol × time” interaction (*p* < 0.001) was found for blood lactate concentration (Figure 2). Although all protocols induced significant increases after exercise, higher VL thresholds resulted in greater lactate concentrations (BFR0 < BFR10 < BFR20 < BFR40).

### 3.4. Mechanical Responses

Mechanical characteristics of CMJ, MVIC, and V1-load tests are shown in Table 3. A significant “time” effect was found for all variables, except for MIF. Significant “protocol × time” interactions were observed for all variables. BFR40 resulted in greater impairments in CMJ height, MIF, and RFDmax at MVIC, and MPF, MPP, and MPV at the V1-load than the other protocols. All protocols induced performance reductions in all variables analyzed, except for RFDmax following BFR10 and BFR20.

### 3.5. Neuromuscular Responses

**Figure 3:**
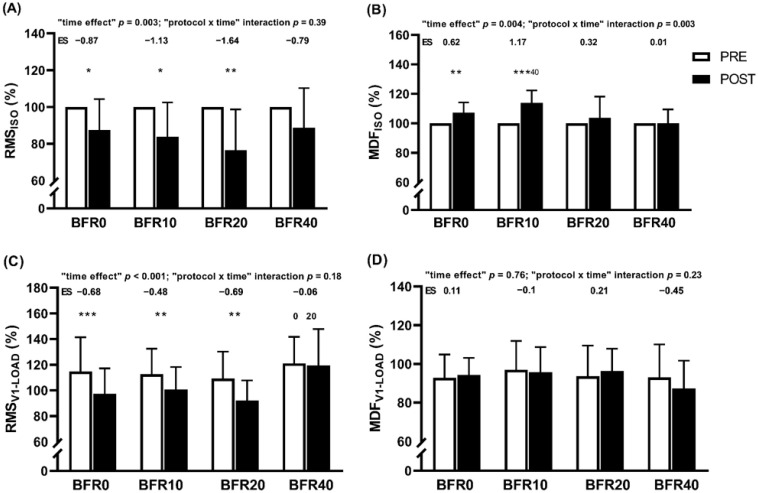
Neuromuscular responses following different resistance exercise protocols with distinct velocity loss thresholds during full-squat training with blood-flow restriction. (**A**) RMS_ISO_: root mean square (RMS) from maximal voluntary isometric contraction (MVIC); (**B**) MDF_ISO_: median frequency (MDF) from MVIC; (**C**) RMS_V1-LOAD_: RMS from the load that elicited a 1 m·s^−1^ at Pre-test (V1-load); (**D**) MDF_V1-LOAD_: MDF from V1-load. BFR0: protocol with a velocity loss of 0% within the set; BFR10: protocol with a velocity loss of 10% in each set; BFR20: protocol with a velocity loss of 20% in each set; BFR40: protocol with a velocity loss of 40% in each set. ES: within-group effect size from Pre- to Post protocol; intragroup significant differences from Pre- to Post protocol: * *p* < 0.05, ** *p* < 0.01, *** *p* < 0.001. ^0^ Statistically significant differences with BFR0 protocol: *p* < 0.05. ^20^ Statistically significant differences with BFR20 protocol: *p* < 0.05. ^40^ Statistically significant differences with BFR40 protocol.

Figure 3 depicts the acute responses in RMS and MDF at MVIC and V1-load tests. A significant “time” effect was noticed for all variables except for MDF at the V1-load test. A significant “protocol × time” interaction was only found for MDF at the MVIC test (*p* = 0.003). BFR10 resulted in higher increases in MDF at MVIC than BFR40. Indeed, only BFR0 and BFR10 induced significant enhancements in MDF at MVIC.

## 4. Discussion

This study is a pioneering attempt to prescribe training volume based on the VL approach in the context of BFR-RE. The main findings of this investigation can be summarized as follows: Increasing VL resulted in more repetitions completed. However, this increase was also associated with decreased mechanical performance and more pronounced neuromuscular (RMS and MDF) alterations within the set. In the post-exercise assessment, higher VL thresholds led to higher impairments in mechanical performance and muscle contractile properties, as well as intensified responses in blood lactate. By contrast, low VL may potentiate neuromuscular activity and muscle contractile properties.

While participants completed more repetitions with increasing VL, the extra repetitions came at the cost of a decrease in the quality of each repetition. The declining mechanical performance is attributed to fatigue development, making it progressively more challenging to produce force and maintain optimal performance [35]. The decrease in performance may be influenced by neuromuscular activity [36]. In this context, our results demonstrated that higher VL thresholds led to increased levels of neuromuscular fatigue within the set, as evidenced by higher RMS and lower MDF, compared to lower VL thresholds. This is consistent with Piqueras-Sanchiz et al. [37], who observed lower MDF values during a long set configuration protocol compared to a shorter set (i.e., 3 × 8 compared to 6 × 4 at 75% 1RM in SQ). The higher EMG amplitude (i.e., RMS) observed for BFR40 may suggest increased muscle activation [38]. This increased activity may result from the recruitment of higher-threshold motor units (MUs), elevated firing frequency, and changes in intrinsic muscle properties aimed at compensating for the force loss that occurs in the fatigued state [39]. Similarly, lower MDF values are typically associated with a decrease in action potential conduction velocity, a reduced firing rate of fatigued fast MUs [39], decreased intramuscular pH [40], and alterations in action potential shape [41]. In this context, the BFR40-induced impairments in mechanical performance may be partially explained by alterations in neuromuscular activity.

This is the first study to examine the alterations induced in muscle mechanical properties by the BFR approach using the TMG device. Our results show that high VL thresholds (i.e., BFR40) exacerbated the exercise-induced impairments in muscle twitch contractile responses (i.e., Dm and Vd). These findings align with those reported by Piqueras-Sanchiz et al. [37], who observed that longer sets led to greater deteriorations in Vd compared to shorter sets in the SQ exercise in a free-flow context. Decreases in Dm and Vd following resistance exercise have been related to fatigue markers such as impaired skeletal muscle function [42], exercise-induced muscle damage [43], and lower activation of the muscle fibers due to increased fatigue [44]. Conversely, only BFR10 induced significant reductions in Tc (“protocol × time” interaction: *p* = 0.02). In general, type II muscle fibers have considerably shorter twitch contraction times than type I fibers [45]. Likewise, a strong relationship has been observed between Tc and fiber type distribution [46]. Accordingly, the shorter Tc observed after the BFR10 protocol may suggest that a higher proportion of fast muscle fibers was activated by the twitch. Therefore, our results show that high VL thresholds exacerbate the impairments in neuromuscular ability and muscle mechanical properties in involuntary actions, while the opposite effect was observed for low VL thresholds.

Interestingly, the data revealed a linear response in blood lactate concentration as VL increases within the training sets (Figure 2). This aligns with previous findings that have reported a strong relationship (r = 0.93–0.97) between VL and blood lactate concentration following free-flow SQ training [20]. Higher lactate concentrations imply a heightened dependence on anaerobic glycolysis for energy production, coupled with a compromised ability to replenish adenosine triphosphate (ATP) and phosphocreatine (PCr) stores [47]. Consequently, sets performed close to failure may lead to a decreased maintenance of PCr stores, elevated metabolite levels in muscles, and partial resynthesis of ATP [47]. The accentuated metabolic stress, along with the altered neuromuscular activity and the compromised mechanical muscle properties, may well explain the more pronounced post-exercise reductions in mechanical performance observed for BFR40 (Table 3). Previous research conducted in SQ with free-flow conditions has also shown that a higher VL threshold (60% 1RM with a 40% VL) induces higher reductions in CMJ height and MPV with the V1-load compared to a lower VL (60% 1RM with a 20% VL) [48]. It is worth noting that low VL protocols (i.e., BFR0 and BFR10) caused significant increases in MDF during the MVIC test and BFR10 exhibited higher values than BFR40 (“protocol × time” interaction: *p* = 0.003). MDF typically rises in proportion to the recruitment of MU with type II muscle fibers [49,50]. Interestingly, this aligns with the discussion on muscle contractile properties, particularly the decrease in Tc noted during the TMG assessment for BFR10, reinforcing the possibility of facilitated recruitment of type II MUs. In this context, another potential explanation may be the occurrence of supernormal muscle fiber conduction velocity, a phenomenon observed during the early recovery phases following isometric contractions, closely linked to an elevated MDF [51]. Regarding the MDF responses during the post-exercise MVIC, there seems to be an inverted U-shaped relationship between VL thresholds and MDF response (Figure 3B). Accordingly, it is necessary to induce a certain level of fatigue during resistance exercise to maximize the neuromuscular response. However, surpassing this threshold by performing additional repetitions may be detrimental to neuromuscular stimuli.

When interpreting our findings, it is important to highlight some limitations. Firstly, the results may not be generalizable to other BFR conditions, such as varying percentages of applied AOP or different cuff widths and materials. Moreover, since the exercise protocol is based on the SQ exercise, it is worth noting that results might vary with alternative exercises (e.g., upper body exercises) or monoarticular exercises, which are typically applied during BFR-RT programs. Additionally, while participants were instructed to adhere to their regular dietary habits and refrain from taking any supplements that might disturb the intervention, the dietary intake throughout the intervention was not quantified.

## 5. Conclusions

High VL in a BFR context (i.e., BFR40) resulted in greater volume. Nonetheless, this increase was accompanied by a decline in mechanical performance and more pronounced neuromuscular alterations within the set. In the post-exercise evaluation, higher VL thresholds induced more profound impairments in mechanical performance and muscle contractile properties, along with intensified responses in blood lactate levels. By contrast, low VL may enhance neuromuscular activity and improve muscle contractile properties.

## 6. Practical Applications

This study offers practical applications for individuals engaged in BFR-RE using SQ. The main application is the use of VL as a metric for tailoring training programs to individual goals, allowing for managing the fatigue induced during resistance training. It is important to note that higher VL thresholds lead to higher training volumes but lower mechanical and neuromuscular quality. Athletes seeking to maximize metabolic stress should use high VL thresholds, though they should be prepared for more significant post-exercise impairments and plan their recovery accordingly. Conversely, low VL thresholds can be integrated into training when seeking low fatigue, potentiated neuromuscular activity, and improved muscle contractile properties.

## Figures and Tables

**Table 1:** Descriptive characteristics of different resistance exercise protocols with distinct velocity loss thresholds during full-squat training with blood-flow restriction.

Protocol	VL (%)	MPF (N)	MPP (W)	MPV (m·s^−1^)	Total Rep (n)	Rep Per Set (n)	RMS (%)	MDF (%)
BFR0	0.0 ± 0.0	838.7 ± 123.7	702.2 ± 112.2	0.91 ± 0.09	3.0 ± 0.0	1.0 ± 0.0	105.7 ± 30.6	96.0 ± 11.8
BFR10	10.2 ± 1.6 ^0^	813.5 ± 128.6	655.6 ± 95.1 ^0^	0.88 ± 0.08 ^0^	13.3 ± 3.7 ^0^	4.4 ± 1.2 ^0^	102.9 ± 17.1	95.7 ± 11.9
BFR20	20.9 ± 2.1 ^0 10^	776 ± 116.1 ^0 10^	585.3 ± 86.9 ^0 10^	0.81 ± 0.08 ^0 10^	22 ± 5.3 ^0 10^	7.3 ± 1.8 ^0 10^	101.4 ± 19.1	93.5 ± 11.4
BFR40	41.4 ± 4.0 ^0 10 20^	727.3 ± 109.1^0 10 20^	501.8 ± 71.3 ^0 10 20^	0.73 ± 0.09 ^0 10 20^	34.5 ± 7.3 ^0 10 20^	11.5 ± 2.4 ^0 10 20^	120.3 ± 23.7 ^0 10 20^	88.5 ± 10.9 ^0 10 20^

Data are mean ± SD. BFR0: protocol with a velocity loss of 0% within the set; BFR10: protocol with a velocity loss of 10% in each set; BFR20: protocol with a velocity loss of 20% in each set; BFR40: protocol with a velocity loss of 40% in each set; VL: average velocity loss attained within the set; MPF: average mean propulsive force attained in each protocol; MPP: average mean propulsive power attained in each protocol; MPV: average mean propulsive velocity attained in each protocol; total rep: total number of repetitions performed; rep per set: average number of repetitions performed per set; RMS: average root mean square attained in each protocol; MDF: average median frequency attained in each protocol; statistically significant differences with BFR0 protocol: ^0^ *p* < 0.05. Statistically significant differences with BFR10 protocol: ^10^ *p* < 0.05. Statistically significant differences with BFR20 protocol: ^20^ *p* < 0.05.

**Table 2:** Muscle contractile properties responses to different resistance exercise protocols with distinct velocity loss thresholds during full-squat training with blood-flow restriction.

	BFR0			BFR10			BFR20			BFR40			Time Effect	Protocol × Time
	Pre	Post	ES	Pre	Post	ES	Pre	Post	ES	Pre	Post	ES	*p*-Value	*p*-Value
Tc (ms)	24.9 ± 4.2	24.0 ± 5.0	−0.19	25.3 ± 3.5	21.6 ± 3.7 ***	−1.01	24.0 ± 3.3	23.3 ± 4.0	−0.19	24.7 ± 4.8	25.5 ± 6.5	0.31	0.10	0.02
Td (ms)	23.8 ± 2.7	21.3 ± 2.8 ***	−0.89	23.7 ± 2.0	20.2 ± 1.9 ***	−1.76	22.8 ± 2.3	20.5 ± 2.0 ***	−1.05	23.7 ± 3.1	21.2 ± 2.6 **	−0.86	< 0.001	0.08
Dm (mm)	5.02 ± 1.71	4.27 ± 1.84 **	−0.41	5.44 ± 1.61	4.27 ± 1.67 **	−0.70	5.16 ± 1.89	3.73 ± 1.16 ***	−0.89	5.54 ± 1.85	3.07 ± 0.91 *** ^0 10^	−1.66	< 0.001	0.03
Vd (mm·ms^−1^)	0.104 ± 0.030	0.092 ± 0.033 *	−0.37	0.110 ± 0.028	0.101 ± 0.033	−0.29	0.107 ± 0.036	0.084 ± 0.025 **	−0.73	0.114 ± 0.034	0.067 ± 0.022 *** ^0 10 20^	−1.61	< 0.001	0.01

Data are mean ± SD; BFR0: protocol with a velocity loss of 0% within the set; BFR10: protocol with a velocity loss of 10% in each set; BFR20: protocol with a velocity loss of 20% in each set; BFR40: protocol with a velocity loss of 40% in each set; Tc: contraction time; Td: delay time; Dm: muscle displacement; Vd: velocity of deformation radial (Dm/(Tc + Td); ES: within-group effect size from Pre- to Post protocol; intragroup significant differences from Pre- to Post protocol: * *p* < 0.05, ** *p* < 0.01,*** *p* < 0.001; ^0^ statistically significant differences with BFR0 protocol: *p* < 0.05; ^10^ statistically significant differences with BFR10 protocol: *p* < 0.05; ^20^ statistically significant differences with BFR20 protocol: *p* < 0.05.

**Table 3:** Mechanical responses following different resistance exercise protocols with distinct velocity loss thresholds during full-squat training with blood-flow restriction.

	BFR0			BFR10			BFR20			BFR40			Time Effect	Protocol × Time
	Pre	Post	ES	Pre	Post	ES	Pre	Post	ES	Pre	Post	ES	*p*-Value	*p*-Value
CMJ (cm)	39.3 ± 6.8	35.6 ± 7.0 ***	−0.56	39.2 ± 6.7	35.0 ± 5.9 ***	−0.63	39.3 ± 6.4	33.5 ± 5.4 *** ^10^	−0.87	39.0 ± 6.7	28.7 ± 5.6 *** ^0 10 20^	−1.55	<0.001	<0.001
MIF (N)	1157.2 ± 159.9	1032.3 ± 153.5 ***	−0.73	1149.5 ± 200.2	1028.5 ± 151.3 ***	−0.71	1146.5 ± 195.3	985.7 ± 152.3 ***	−0.94	1077.7 ± 155.3	912.2 ± 138.9 *** ^0 10^	−0.97	0.12	<0.001
RFDmax (N·s^−1^)	4478.8 ± 1189.9	3946.9 ± 1103.7 **	−0.43	4112.5 ± 1197.7	3840.5 ± 1230.5	−0.22	4015.4 ± 1463.2	3775.7 ± 1407.9	−0.19	4078.4 ± 1068.7	3076.6 ± 960.2 *** ^0 10 20^	−0.80	<0.001	<0.001
MPF-V1 (N)	867.7 ± 133.8	821.6 ± 132.9 ***	−0.35	860.4 ± 132. 9	834.1 ± 135.7 ***	−0.20	855.5 ± 129.9	826.2 ± 124.9 ***	−0.22	852.4 ± 132.4	789.7 ± 128.3 *** ^0 20^	−0.48	0.05	<0.001
MPP-V1 (W)	761.5 ± 129.4	665.0 ± 117.9 ***	−0.84	752.2 ± 119.8	690.1 ± 106.7 ***	−0.54	754.3 ± 117.4	673.2 ± 96.2 ***	−0.70	753.8 ± 121	590.1 ± 113.3 *** ^0 10 20^	−1.42	0.006	<0.001
MPV-V1 (m·s^−1^)	0.98 ± 0.09	0.88 ± 0.09 ***	−1.18	0.96 ± 0.09	0.89 ± 0.07 ***	−0.71	0.97 ± 0.06	0.88 ± 0.07 ***	−1.18	0.98 ± 0.07	0.80 ± 0.09 *** ^0 10 20^	−2.13	0.004	<0.001

Data are mean ± SD; BFR0: protocol with a velocity loss of 0% within the set; BFR10: protocol with a velocity loss of 10% in each set; BFR20: protocol with a velocity loss of 20% in each set; BFR40: protocol with a velocity loss of 40% in each set; CMJ: countermovement jump height; MIF: maximal isometric force: RFDmax: maximal rate of force development; MPF-V_1_: mean propulsive force of the best attempt with the load that elicited a 1 m·s^−1^ at Pre-test (V1-load); MPP-V_1_: mean propulsive power of the best attempt with the V1-load; MPV-V_1_: mean propulsive velocity of the best attempt with the V1-load; ES: within-group effect size from Pre- to Post protocol; intragroup significant differences from Pre- to Post protocol: * *p* < 0.05, ** *p* < 0.01,*** *p* < 0.001; ^0^ statistically significant differences with BFR0 protocol: *p* < 0.05; ^10^ statistically significant differences with BFR10 protocol: *p* < 0.05; ^20^ statistically significant differences with BFR20 protocol: *p* < 0.05.

## Data Availability

The data showed in this study are available on request from the corresponding author. The data are not publicly available due to containing information that could compromise the privacy of research participants.
